# Perspectives on human-human sensorimotor interactions for the design of rehabilitation robots

**DOI:** 10.1186/1743-0003-11-142

**Published:** 2014-10-06

**Authors:** Andrew Sawers, Lena H Ting

**Affiliations:** Wallace H. Coulter Department of Biomedical Engineering, Emory University and Georgia Institute of Technology, 313 Ferst Drive NE, Atlanta, 30332 USA

**Keywords:** Rehabilitation, Human-human interaction, Human-robot interaction, Haptics, Rehabilitation robotics

## Abstract

Physical interactions between patients and therapists during rehabilitation have served as motivation for the design of rehabilitation robots, yet we lack a fundamental understanding of the principles governing such human-human interactions (HHI). Here we review the literature and pose important open questions regarding sensorimotor interaction during HHI that could facilitate the design of human-robot interactions (HRI) and haptic interfaces for rehabilitation. Based on the goals of physical rehabilitation, three subcategories of sensorimotor interaction are identified: *sensorimotor collaboration*, *sensorimotor assistance*, and *sensorimotor education*. Prior research has focused primarily on sensorimotor collaboration and is generally limited to relatively constrained visuomotor tasks. Moreover, the mechanisms by which performance improvements are achieved during sensorimotor cooperation with haptic interaction remains unknown. We propose that the effects of role assignment, motor redundancy, and skill level in sensorimotor cooperation should be explicitly studied. Additionally, the importance of haptic interactions may be better revealed in tasks that do not require visual feedback. Finally, cooperative motor tasks that allow for motor improvement during solo performance to be examined may be particularly relevant for rehabilitation robotics. Identifying principles that guide human-human sensorimotor interactions may lead to the development of robots that can physically interact with humans in more intuitive and biologically inspired ways, thereby enhancing rehabilitation outcomes.

**Electronic supplementary material:**

The online version of this article (doi:10.1186/1743-0003-11-142) contains supplementary material, which is available to authorized users.

## Introduction

Designing robots that can physically interact and move with humans to cooperatively perform motor tasks, physically assist in achieving motor goals, or aid in rehabilitation of movement is a grand challenge of robotics. Robots have the potential to improve rehabilitation by initiating treatment earlier than may otherwise be possible, increasing the intensity of training, creating enriched environments that simulate real-world conditions, and allowing patients to practice motor tasks that they may otherwise be unable to perform alone. However, for robotics to successfully serve such roles in rehabilitation, the interaction between robot and patient should be one that is intuitive and natural [1]. Many previous studies have examined physical interactions between humans and robots in an effort to determine how such robots should be controlled [2, 3] and what roles they should adopt [4, 5] to train [6–8], collaborate with [9], or assist [10] humans in an intuitive and natural fashion [1]. These approaches generally seek to identify desirable features based on intuitive notions of what would work [11]. As a result, rehabilitation robots have largely been implemented in an ad-hoc fashion [12] based on classical control methodologies. For example, many rehabilitation robots are controlled by specifying desired kinematic trajectories that are enforced by moving a subject’s joints along a fixed kinematic path [13]. Other control schemes provide assistance on an assist-as-needed basis, (i.e. when subjects deviate from a specified path) [14–17]. Recent evidence in stroke and spinal cord injury suggest that rehabilitation robots that overly constrain joint motions, are only equal or less effective than more traditional types of movement therapies [18, 19]. In contrast, recent advances in human motor learning suggest that humans learn more when errors are larger [20, 21] or when movement variability is greater [22, 23]. This has sparked a new approach in the control of rehabilitation robots; to augment rather than reduce errors [24, 25] (i.e., or to allow patients to explore new movement strategies [26, 27]. Alternatively, rather than providing assistance or resistance to reduce movement or augment movement errors for an entire kinematic trajectory, rehabilitation robots could be designed to provide assistance or resistance to particular portions of a movement. They may also target specific muscle groups that are impaired as a result of injury or disease [28–30]. The differences in these competing hypotheses about sensorimotor learning have direct implications for how best to design robots that physically interact with humans in a rehabilitation context.

A complementary approach to augment the engineering approaches described above is to identify relevant sensorimotor principles of human-human interactions (HHI) that could guide the design and control of rehabilitation robots. It has been suggested that rehabilitation robots based upon principles of human-human sensorimotor interaction [31] would interact with humans in a flexible and intuitive way [32, 33]. Such designs may have the advantage of minimizing the training required to effectively use them [34, 35]. Despite the successes in developing robots that interact with humans physically, it is not clear the degree to which interacting with a robot invokes the same sensorimotor strategies that would be used in the absence of the robot (i.e. alone) or when interacting with another person. One reason is that interactions with a robot are largely driven by the design of the controller itself. Therefore, the success of the interaction is based upon the human’s ability to adapt and learn how to best interact with the robot. While understanding how humans physically interact with each other may be of substantial importance to improving the design of robots that will interact with humans [36], sensorimotor interactions between two or more humans are poorly understood from a scientific perspective.

In this review we define HHI to be any sensorimotor interaction that occurs between two or more physically connected individuals. Participants do not necessarily need to be working towards a common motor goal, but in its application to physical rehabilitation this would generally be the case. Bimanual interactions performed by a single individual could be viewed as an additional form of motor interaction. In fact, bimanual interactions share similarities with HHI including the use of specialization strategies to coordinate the actions of each arm during motor performance [37]. However, bimanual interactions include neural coupling between the two arms, while interactions in HHI must take place exclusively through haptic or other sensory channels between participants. As a result, the exchange and interpretation of shared information differs fundamentally between HHI (*two brains, two hands*) and bimanual interaction (*one brain, two hands*). Therefore, this review will focus on physical interactions between two humans with consideration to the perspective of what can be gained for understanding rehabilitation robotics. Readers are encouraged to seek out previous reviews of bimanual interaction [38–40] to supplement the current review on HHI. Similarly, while relevant to the larger dialog on rehabilitation robotics, an extensive discussion of HRI is beyond the scope of the current manuscript. Readers are encouraged to consider other excellent works on this topic to supplement the current review of HHI [12, 41].

To address the gap in our understanding of fundamental principles governing HHI, here we outline areas of study in HHI that may be useful for guiding the design and use of rehabilitation robots. We begin by reviewing proposed taxonomies of HHI, and evaluating their application to physical rehabilitation. We then examine the existing HHI literature, analyzing the principles of HHI that have been elucidated to date, and the experimental paradigms and metrics used in their identification. Finally, we highlight important future directions and unanswered questions about the nature of physical interactions between human partners and how they may reveal general principles of HHI relevant to HRI in the context of rehabilitation.

## Review

### Taxonomies of human-human sensorimotor interaction in the context of physical rehabilitation

There is currently no widely accepted framework for understanding HHI in the context of its application to rehabilitation robotics. Here we will review the different ways in which HHI have been conceptualized, and elaborate on aspects of HHI classification with respect to the objectives of physical rehabilitation, the roles assigned to participants, and their relative skill level.

As early as 1956, Wegner [42] proposed an initial framework for conceptualizing the factors influencing HHI. Wegner [42] identified a list of categories of aggregate organism or group behavior including: cooperation, social facilitation, guidance, leadership, problem solving, communication, learning, and transfer. These categories largely classified the factors that could improve HHI performance in the context of a visuomotor tracking task. Relevant to this review, they provided data to support three points as follows.

1. The greater the number of participants performing simultaneously, the higher the performance level (*social facilitation*).
2. The transfer of performance from one team size to another varied (*learning and transfer*).
3. The most important determinant of team performance was either the skill of the best solo performer (in quads) or the worst solo performer (in dyads) (*leadership*) [42].

While all of these questions are germane to the questions facing rehabilitation roboticists, their approach did not examine how the interactions actually occur. This is critical for translating of findings to the design and use of robots that could participate in similar interactions.

More recently, HHI has been classified according to the emergence of specific roles adopted by participants and the resulting interaction forces. Melendez-Calderon [43] used interaction forces and muscle activity in order to infer the various roles that could be adopted in a task. In their example, the motion of each participant’s wrist is coupled via a physical linkage and subjects must jointly move their wrist to drive a cursor to a target on a screen. They identified roles that ranged from both agents in the dyad performing overlapping functions, to various combinations of specialization, where each agent in a dyad performed specific portions of a movement [43]. In this study, there was no *a prior* i role assignment in the partnered visuomotor task. As a result, the roles described are adopted spontaneously and often changed over time. However, it is not clear that such emergent roles are immediately applicable to physical rehabilitation, where roles are typically assigned a priori and generally remain fixed.

Powell and O’Malley [44] adopted a more rehabilitation-centric approach by describing a classification scheme of haptic interactions based on *a priori* role assignment. Here, one member of a dyad is consistently directing the movement of another. They proposed that three factors could be used to classify the resulting motor interactions:

1. the type of interaction force applied (i.e. assistive or resistive);
2. how task forces, those generated from the dynamics of the task, are differentiated from externally applied guidance forces, and
3. how the weights of these two types of forces vary over time with respect to motor performance [44].

This approach provides important insights into the characterization of physical interactions within the context of physical rehabilitation and the differential role that each member of a dyad can adopt. One aspect of the interactions that is not considered is how they may differ depending on the goals associated with the motor interaction. For example, the forces may vary depending on whether the leader is providing assistance for a motor deficit, or aiming to train an individual to improve their motor ability beyond the partnered condition.

Jarrasse [36] proposed a comprehensive framework for classifying motor interactions based on minimizing error and effort in each participant [36]. This is a common approached used in predicting motor behaviors with optimal control theory [45, 46]. Different classes of motor interaction were defined by the appearance of the error and effort in the objective function that describes the nature of the task as well as the combination of each agent’s behavior. The proposed taxonomy classifies sensorimotor interactions into three main categories: *competition*, *collaboration* and *cooperation*. During competitive sensorimotor interactions both participants consider only their own effort and error. Such interactions typically emerge during the performance of antagonistic tasks, as exemplified by sports such as wrestling, where the gain of one agent results in loss of the other agent. In contrast, during collaborative and cooperative sensorimotor interactions, each participant considers there own effort and error as well as that of there partner, attempting to work together to identify a mutual beneficial resolution to a task. Therefore, unlike competitive interactions, collaborative and cooperative interactions occur in the performance of agonistic tasks, where gains and losses similarly affect both agents in a dyad, such as in rowing. Collaborative and cooperative interactions differ based on how roles are assigned to each participant and how that impacts their contribution to the interaction. In collaborative interactions there is no a priori role assignment. Roles are adopted in a spontaneous manner and subject to change. Emerging from this is an equal distribution of responsibilities or work between the participants. In contrast, during cooperative interactions roles are assigned a priori to each subject, and these roles are maintained throughout the performance of the interactive task. This creates asymmetric interests and an uneven distribution of sub-tasks between the participants such that while both individuals are working towards the same goal, they are doing so by performing different parts of the same task.

**Figure 1:**
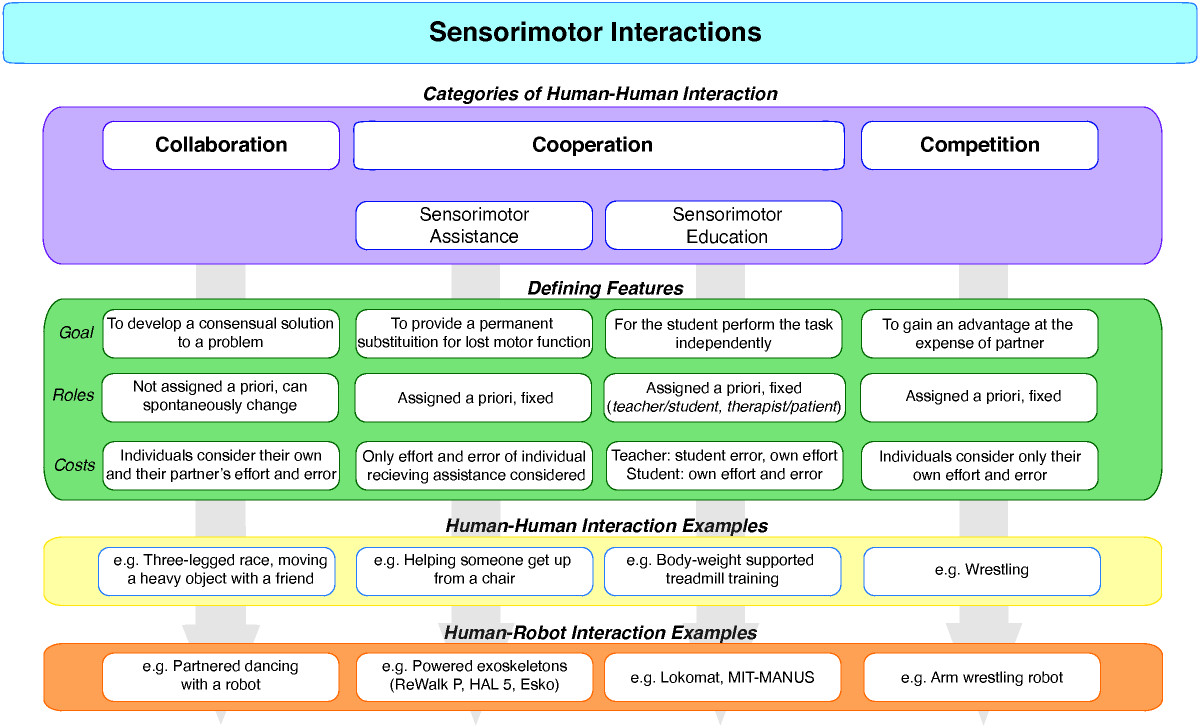
**Taxonomy of sensorimotor interactions based upon the classification proposed by Jarrasse** [36]**.** Each category of human-human interaction (HHI) can be classified based on several defining features including: its goal, how the roles of each member of the dyad are determined, and the cost(s) that each member attends to. HHI and human-robot interaction examples are provided illustrate differences between categories.

*Assistance* and *education* are two forms of cooperation identified by Jarrasse [36] that are particularly relevant to rehabilitation. During assistance, both members of a dyad are only concerned with the effort and error of the individual who is receiving assistance. This is akin to a robotic exoskeleton being used to perform a motor task where the effort of the exoskeleton is not considered. We would extend assistance to HHI, where one participant is providing assistive forces, cues, or corrections to a second participant to achieve a motor goal that the second participant may not be able to accomplish on his or her own (e.g. getting up from a chair). In this case, haptic cues and interaction forces may be used to monitor a participant’s need for assistance as well as to deliver that assistance. During education, a teacher aims to reduce the error of the student while minimizing their own effort, whereas the student is only concerned with minimizing his or her own error and effort. The goal of education is for the teacher to eventually become obsolete, allowing the student to perform the task independently [36]. From the perspective of physical rehabilitation, education most resembles that of a therapist training a patient in the clinic to improve their motor performance beyond the clinic (i.e. at home or in the community). We believe that an important distinction between assistance and education is that assistance represents an end-solution to motor impairment such as provided by an assistive device, rather than an attempt to improve independent motor performance beyond the partnered condition, as in education (Figure 1).

One important difference across these taxonomies is the ways in which the roles of the participants are defined. In the taxonomy proposed by Jarasse [36], roles are based on the objective function of each participant. In contrast, in experimental work reviewed below, the role(s) of each participant are inferred from the interaction forces that emerge during a HHI task where there was no explicit a priori role assignment [43]. Finally, in some cases, roles are defined a priori by which participants are expected to direct the other, and the manner in which they do so [44]. Such a priori role assignment could be explained by asymmetries in the objective functions of the participants, but these may differ from the specific objective functions proposed by Jarrasse [36]. For example, recent human sensorimotor research suggests that learning may be increased when errors are augmented [25, 47, 48] or when performance is more variable [22, 23, 49], not when errors are minimized. As a result, in contrast to an error minimization objective, a therapist may wish to augment errors by resisting the actions of a patient, thereby increasing rather than decreasing error. Further, a priori role assignment could also occur in collaborative interactions in which the goal is neither education nor assistance. For example, in partner dance the leader and the follower may have different objective functions where the leader may care about his own error as well as the error of the follower, and the follower only attends to his or her own error. Nevertheless, the classification scheme proposed by Jarasse [36] is a reasonable starting point for beginning to understand how HHI is relevant to rehabilitation robotics.

### Prior work relevant to human-human sensorimotor collaboration

Of the three forms of HHI behavior defined by Jarasse [36], cooperative interactions predominate in rehabilitation (*i.e. education and assistance*). Here, leader-follower or teacher-student roles are predefined. However, the vast majority of the prior research involving HHI has focused on sensorimotor *collaboration.* Here, two individuals share a common goal, but have no pre-defined roles*.* Next we review prior studies in HHI, discussing the experimental paradigms, performance metrics, and the influence of haptic information during sensorimotor collaboration between two human partners (Table 1).

**Table 1:** **Overview of the interactions used to study human-human interaction to date and the resulting outcomes**

Principal findings	Papers
***Experimental paradigms***	
1. HHI research has predominantly focused on sensorimotor collaboration, not cooperation	
*Sensorimotor collaborations*	Reed [56, 65], Groten [70], Melendez-Calderon [43], Ikeura [50], Rahman [51], Basdogan [52], Sallnas [53], Gentry [54, 64], van der Wel [55], Feth [66]
*Sensorimotor cooperation*	Ikeura [60], Galvez [86]
2. The majority of HHI research has used visuomotor tasks with limited degrees-of-freedom	
*Constrained visuomotor tasks*	Melendez-Calderon [43], Ikeura [50], Rahman [51], Basdogan [52], Sallnas [53], Gentry [54, 64], van der Wel [55], Reed [56]
*Whole-body non-visuomotor tasks*	Galvez [86]
3. In most HHI research, specific roles for each member of a dyad are rarely defined ahead of time	
*Unassigned roles*	Reed [65], Groten [70], Melendez-Calderon [43], Rahman [51], Basdogan [52], Sallnas [53], Gentry [54, 64], van der Wel [55], Reed [56, 65], Feth [66]
*Assigned roles*	Ikeura [50, 60]
***Experimental outcomes***	
4. Dyads typically perform as well or better than either member of a dyad alone	
*Superior dyad performance*	Reed [65], Gentry [54], Feth [66]
*Equivalent individual and dyad performance*	van der Wel [55]
5. The addition of haptic feedback improves dyad performance compared to visual feedback alone	
	Basdogan [52], Sallnas [53], Gentry [64], Groten [67, 71]
6. Members of a dyad apply higher forces than during either of their individual performances	
	van der Wel [55], Reed [68], Groten [70, 71], Feth [66]
7. Members of a dyad spontaneously assume specific roles, performing portions of a joint motor task	
	Melendez-Calderon [43], Reed [32, 56]
*Starting or ending movement*	Reed [32, 68]
*Adding or absorbing energy*	Feth [66]

#### Prior paradigms for sensorimotor collaboration

*Sensorimotor collaboration* tasks are often motivated by whole-body motor tasks such as lifting a heavy object or folding a bed sheet with a friend. However, the study of sensorimotor collaboration has focused primarily on visuomotor tasks that require limited degrees-of-freedom. These joint motor tasks include real and virtual object manipulation [50–53], trajectory tracking, and target acquisition [43, 54, 55]. Participants typically sit across from each other or face a computer screen while holding a manipulandum that provides a haptic link which can provide a direct physical [56] or virtual coupling [52]. While visual communication and observation of the other participant is usually obscured, participants rely on visual feedback to complete the motor tasks in these paradigms. Participants are given a visual signal to complete the task as quickly or as accurately as possible. For both physically and virtually linked dyads, the tasks are such that they can be performed either individually, or as a dyad, and the performance can then be compared.

Limiting the study of collaborative HHI to motor activities that require visual input *a priori* may confound our understanding of the role of haptic feedback in driving motor performance. In all of the prior tasks, visual information is used to define the success and failure of the task and to provide feedback about performance. Haptic information is only secondary in these tasks, providing information about the physical interaction or coupling with another person, but not about the achievement of the task goal. While the influence of haptic information in addition to visual feedback can be identified, the role of haptic information alone in performing tasks remains unknown. In HHI, visual feedback has been shown to induce coupling between members of a dyad for rhythmic coordination tasks [57–59]. Further, the inclusion of visual feedback during HHI has been shown alter how the two agents in a dyad contribute to task performance [60]. However, there are many tasks that may be primarily haptic in nature as somatosensory information may be processed more quickly and with better resolution than visual information [61]. Even basic HHI tasks such as holding someone’s hand during a handshake or leading someone across the street provide haptic feedback as the participant’s vision is typically focused elsewhere.

While discrete, limited degree-of-freedom tasks are studied for their experimental tractability, sensorimotor coordination and learning principles derived from such movements do not necessarily generalize to complex, multi-joint movements that are often the focus of rehabilitation [62]. For example, the control of walking, a common focus of rehabilitation, has a very different sensorimotor and neural organization compared to reaching [63]. Further, one hallmark of the flexibility and robustness of the human motor system is the inherent redundancy of the motor behaviors at both the kinematic and muscular levels. This may be reduced when studying single degree-of-freedom tasks. The variations in how motor tasks can be achieved may play an important role in the haptic information that is conveyed between individuals.

Finally, with a few exceptions [50, 60], the specific role for each member of a dyad in prior paradigms is rarely assigned ahead of time. The specific roles of each individual are left to emerge and allowed to change during the course of a trial or an experimental session. These roles are inferred from the forces measured in the task, and the factors affecting the emergence of different roles that people adopt are unknown, and may depend upon their understanding of the task, motivation, skill, or strength. These inferred roles and measured forces might not correspond with forces generated when participants knowingly assume a role. Therefore, HHI based on unassigned roles may have a limited application to physical rehabilitation, where roles are typically assigned and maintained during training.

#### Prior metrics used in sensorimotor collaboration

A variety of kinematic metrics have been used to assess the motor performance of individuals and dyads during collaborative visuomotor tasks. The position, velocity, and acceleration of the objects (virtual or real) through which members of a dyad interact are sufficient to define the kinematics of the task [32, 50, 51, 55, 64]. Based on kinematics, performance on collaborative visuomotor tasks has been evaluated using a variety of temporal [52, 54, 55, 65], and spatial accuracy measures [52–55, 66–68]. These include time to complete task, time on target, and tracking error. While these measures are helpful in describing the outcome of an interaction, they yield limited insight into the sensorimotor processes used by each member of a dyad to achieve that outcome.

Various kinetic metrics have also been used to characterize HHI, often as a means to describe the contributions of each member in a dyad to the interactive visuomotor task. Ikeura [60] examined the work performed by each member of the dyad during a shared lifting task, while Rahman [51] assessed the correlation between the forces exerted by each subject and the resulting acceleration of a shared object. Other authors have attempted to identify different roles or strategies that members of a dyad adopt during collaborative motor interactions by examining differences in force magnitude between dyad and solo performance [32], the degree to which the forces each member of the dyad exert overlap, and how this overlap varies with the phase of the collaborative motor task [55]. One of the challenges in using force information to deduce roles is the fact that forces result from the dynamic interactions between individuals and cannot be independently interpreted. First, reaction forces that are not explicitly generated by muscles or any particular control strategy can result based on the kinematic constraints of a movement [69]. Therefore, in the context of HHI, the actions or forces of one individual are dependent upon those of the other. To generate a force when one person pushes requires that the other person resist. Such a response could be dependent upon relative reaction time and strength in the kinds of HHI tasks previously studied.

More complex constructs such as effort and efficiency [67, 70, 71] have been examined through combinations of kinematic and kinetic measures. Interestingly, more common measures of efficiency and effort in sensorimotor control such as metabolic cost [72], and movement smoothness [73] have not been used. Recent advances in sensorimotor control theory suggest that movements result from a tradeoff between measures of effort and performance as described by optimal control theory [45, 46, 74, 75]. While included in the motivation for a taxonomy of HHI [36], optimal control theory has not yet been directly applied to the study of HHI. Such a framework may also prove useful to compare results from HHI and HRI experiments.

More recently, muscle activity has been recorded [43] as a way to understand the motor strategies used by the nervous system during HHI. Muscle activity can provide a more nuanced, measure of the different motor strategies used by participants, as well as their perception of the actions of their partner. There is also redundancy in both the muscles that can be used to perform a task, as well as the temporal patterns of muscle activity to achieve a particular kinematic trajectory [76, 77]. Although challenging to study, muscle activity can provide a window into the sensorimotor processes used for generating movements [77–80].

#### Two is better than one: HHI improves task performance

Dyads that are haptically linked can perform collaborative visuomotor tasks as well as [55] or faster than [32, 54, 65], and with greater accuracy [66] than either member of a dyad alone. In spite of this improvement in performance, the haptic interaction is perceived as an impediment by individual members of a dyad when compared to solo performance [32]. Reed [65] reported that these improvements in motor performance among dyads do not occur instantaneously, but rather within 20 trials. This indicates that some time is necessary for members of a dyad to learn how to communicate and use available haptic interactions. This may be particularly true as task difficulty increases [54]. Additionally, the performance improvement due to haptic interaction may depend on an individual’s expertise to integrate haptic information or their initial motor skill level [81]. Knoblich [82] suggested that improvements in performance are due to the fact that as a dyad, individuals have fewer actions to deal with, allowing them each to focus on a subset of actions.

In early HHI studies, social facilitation was suggested as a potential explanation for performance improvements in dyads versus solo performance [42]. It is well established that merely having others around or nearby encourages participants to try harder, facilitating motor performance [59, 83]. But, in more recent experiments, the presence of the researcher may have contributed to social facilitation during both solo and dyad performance. However, social facilitation as an explanation for performance improvement amongst dyads has not been explicitly tested.

#### Haptics improve performance of dyads during sensorimotor collaboration

In dyad performance during sensorimotor collaboration, the addition of haptic feedback improves visuomotor task performance compared to conditions where only visual feedback is available [52, 53, 64, 71]. As with solo versus dyad performance, some time is required after the addition of a haptic interaction before improvements in performance are seen. This is especially the case with more complex collaborative visuomotor tasks [67, 71]. Additionally, haptic interaction appears to improve dyad performance more among younger versus older adults, and in females versus males [52]. This suggests the potential importance of considering individual needs and experiences when designing and implementing haptic interfaces in rehabilitation robotics. Also, in contrast to the haptic interaction being considered an impediment when going from solo to dyad performance [55], members of dyads report that the addition of haptic interactions during a visuomotor task increases the sense of “togetherness” [52]. Improved performance with the addition of a haptic interaction is consistent with the faster speed of haptic versus visual information. Haptic feedback is potentially twice as fast as visual feedback [84], and also relies on changes in forces, which precede position or velocity errors that are eventually perceived by the visual system. Since visual feedback alone can induce substantial coupling between members of a dyad during rhythmic coordination tasks [57, 58], limiting the study of sensorimotor collaboration to motor tasks that require visual input *a priori* may confound our understanding of haptic interaction in cooperative HHI and HRI.

#### Elevated interaction forces may facilitate collaborative motor task performance

Performance improvements by haptically linked dyads in visuomotor tasks are accompanied by alterations in the forces between participants. During visuomotor task performance, both members in a dyad apply higher forces than during either of their individual performances [68]. This occurs despite similar levels of performance error [85]. Non-zero interaction forces characterize haptically linked dyads [55], although their magnitude varies greatly across dyads [70], and increases during faster movements as well as during different phases of movement [55]. Given that interaction forces decrease mechanical efficiency [67, 70], increase mechanical effort [67, 71], and do not contribute directly to visuomotor task performance, their role in accomplishing collaborative motor tasks has been a source of much speculation.

Originally, the opposing forces (*e.g. pushing or pulling*) between individuals in a haptically linked dyad were thought to stabilize HHI [68, 71], much in the same way that the co-contraction of antagonistic muscles can stabilize a joint. However, the elevated interaction forces did not improve stability to external perturbations, as haptically linked dyads were less effective in resisting physical perturbations than individuals [56]. Alternately, an increase in interaction forces may arise from an increase in arm stiffness. This could “prime”, or facilitate, the sensorimotor system by increasing its sensitivity to sensory inputs caused by the movements between partners [55, 56, 71] or by pre-activating muscles, facilitating faster motor responses [66]. However, the degree to which maintaining some minimum interaction force actually improves sensorimotor performance in collaborative motor tasks during HHI remains unknown.

#### Role specialization emerges during sensorimotor collaboration

Performance improvements in collaborative HHI have also been attributed to the adoption of specialized roles by the members of haptically-linked dyads [65]. Rather than assuming responsibility for the entire task [43], participants appear to focus on executing specific portions of a motor task such as starting or ending the movement [32, 68], or adding versus absorbing energy [66]. The roles assumed by members of a dyad have been shown to vary over different phases of a movement [56], and change over time [43]. In each of these cases where role specialization has been reported, participants were engaged in interactive motor tasks where they were not assigned roles a priori (i.e. collaboration). Therefore, a reasonable and open question to pose is how and why do these specialized roles develop? It is unclear whether these strategies emerge due to conscious decisions made by members in a dyad as a way to reduce their own effort and/or that of their partner. It is possible that their emergence is due to differences in strength, reaction time, attention or skill level between agents in a dyad. If such factors are influencing the development of these specialized roles, then interesting questions begin to emerge regarding how individuals should be chosen and assigned to a dyad. One may be able to influence the performance and behavior of each member of a dyad, as well as the dyad as a whole simply by considering these psychomotor characteristics when assigning individuals to a dyad.

## Discussion

### Open questions and future directions regarding principles of sensorimotor cooperation

While the work summarized above has begun to provide some valuable insight into aspects of HHI, general principles that could be translated to the design and use of rehabilitation robots have not yet been identified. The field of HHI is relatively young. As such, the results reviewed above must be confirmed through additional studies aimed at testing specific hypothesis about sensorimotor cooperation, particularly in the context of sensorimotor assistance and education. While exploratory studies are necessary to characterize interactions, detailed and controlled experiments designed to isolate the role of specific features of the interaction are necessary. Thus hypotheses must be systematically tested against control conditions over a variety of different cooperative motor tasks in order to identify general principles of sensorimotor cooperation. Here we pose several unanswered questions about HHI to frame further experiments in sensorimotor cooperation. These considerations may help identify novel experimental paradigms to study cooperative HHI, which in turn may reveal important principles of haptic interaction relevant to the design and use of rehabilitation robotics.

#### How should haptic feedback be used to promote motor education?

While cooperative sensorimotor assistance and education serve as the motivation and basis for a large number of therapist and robot-based rehabilitation interventions, almost no literature exist examining the force interaction between individuals in these two contexts. For example, physical therapists regularly provide haptic feedback to patients by physically guiding their movements during practice. However, little is known regarding how those forces should be applied to encourage motor skill learning (i.e. cooperative sensorimotor education). In a single study examining the forces that physical therapists apply during sensorimotor training, Galvez [86] found that experienced physical therapists each applied substantially different forces to individuals with spinal cord injury during body-weight supported treadmill training, resulting in different leg kinematics. How these differences in technique translated to the generalization of locomotor performance beyond treadmill training was not reported. Ikeura [60] also examined the force characteristics during a sensorimotor assistance task, although one that is more practical experimentally than applicable clinically, lifting and moving a light object over a 15-centimeter distance. As with Galvez [86], the objective here was not to examine how force interactions contributed to task performance, but rather to characterize the forces used during the task in order to develop a controller that would mimic the behavior of two humans [60].

In future work it may be helpful to verify that elevated interaction forces facilitate the observed improvements in motor performance during dyad versus solo performance. To further probe the role of elevated interaction forces between human partners and how it may influence motor performance, one could imagine a simple experiment where several different conditions are tested. In each condition the initial interaction force (compressive and tensile) between the human partners is systematically varied. Motor performance from each condition could then be compared to examine the role of the elevated forces that have been observed during HHI.

In contrast to the work of Galvez [86], which focused on characterizing the force interaction, most studies of cooperative sensorimotor education look at improvements in performance without considering the interaction forces that arise between the patient and therapist or coach. Generally, sensorimotor education, or physical guidance, has been shown to improve immediate performance of simple motor tasks, but these improvements are typically not retained nor generalized to individuals performance [87–89]. The application of constant haptic guidance may suppress the development of error detection and correction processes, creating dependency on the haptic guidance for improved motor performance [90]. Constant haptic guidance may also limit movement variability, which is thought to be important to motor learning for its role in allowing participants to explore the space of possible movements [22, 23]. The generalization of performance improvements from partnered to individual conditions following sensorimotor training may be more effective for complex, whole-body motor tasks [91] that are more challenging [92], particularly when aspects of safety pose particular dangers (*e.g. body-weight supported treadmill training*). In such situations, haptic guidance via cooperative sensorimotor education may increase patient confidence and limit safety concerns, providing individuals the opportunity to identify not only the most effective, but also the most efficient movement strategies.

Thus there remains a substantial gap in our understanding of how haptic feedback should be used during sensorimotor education to correct movement errors with the goal of promoting skilled individual performance. Currently we lack a framework and experimental data describing how humans physically interact, much less how therapists physically guide patients during rehabilitation. As a result there is not a solid scientific understanding of how this can best be achieved or what principles roboticists should attend to when designing robots for the purposes of rehabilitation.

#### How does role assignment influence sensorimotor cooperation?

Examining how the assignment of specialized roles influences HHI may be of particular relevance to rehabilitation robotics. During physical rehabilitation, therapists initially take on the role of leader by physically guiding the movements of the patient, who in turn takes on the role of follower. As motor recovery progresses, those roles may be altered, with the therapist assuming less and less of a leader role and the patient attempting movements independently or driving the learning. However, the difference in the role of leader versus that of follower in cooperative sensorimotor HHI among unimpaired adults is not well understood. Therefore, future research should be explicit about the roles of the participants in order to accurately represent motor interactions between therapist and patient (*i.e.* more *cooperative and* less *collaborative interactions*). Clearly defining the roles for each member of a dyad when studying cooperative HHI may allow for a more specific characterization of how the use of haptic information differs across the roles of leader and follower. It may also simplify data analysis, and provide specific insight regarding how a robot should emulate a leader versus a follower for the purposes of physical rehabilitation. Specifically, a more detailed analysis of the roles of dominance as examined through HHI may prove to be particularly relevant to identifying principles that could inform assist-as-needed control strategies implemented in rehabilitation robotics. As a patient’s performance improves, and the therapist’s role as leader decreases, with fewer or smaller errors requiring correction, what changes take place in regards to the interaction forces between them? Does the magnitude of the force decrease? Does the consistency with which forces are applied change? By examining how role assignments change over time with improvements in motor performance during HHI, we may develop a better understanding of how force interactions between humans and robots should be modeled in rehabilitation robotic control schemes. Interestingly, individuals in a haptically linked dyad prefer to interact with some dominance difference [33], suggesting that role assignment is an important component to the design of bi-directional haptic interfaces in rehabilitation robotics.

#### How do cooperative sensorimotor interactions vary across skill level?

Cooperative sensorimotor interactions may vary significantly with the skill level of the participants. This is of particular interest in rehabilitation where the therapist is considered an expert, and the skill level of the patient will vary as a function of his or her level of impairment. The study of skill level is complicated by the fact that expertise is commonly understood to take a considerable amount of time to acquire [93]. Therefore, it may not be reasonable to expect adequate skill acquisition to occur over a relatively short period of an experiment. It may be beneficial to select cooperative motor tasks in which there are established expert performers that can be easily identified and recruited. Studying individuals ranging from novices to experts can help to identify a continuum of performance that may provide insight into how rehabilitation robots should interact “expertly”, and to discern the skill of the patient based on haptic interactions.

#### How do participants co-adapt during cooperative sensorimotor interactions?

While interaction forces have been observed to change during cooperative HHI [43, 67], it is not known how participants adapt their movements based on haptic feedback in order to achieve the cooperative task goal(s). The notion of dual-learning or co-adaptive behavior [94] over short and long-term sensorimotor cooperation should be considered. The importance of this co-adaptation is consistent with the finding that simply playing back a recorded trajectory of human movement as the second member of a dyad is not sufficient to generate dyad performance equivalent to that of two human participants [13, 65]. The manner in which individuals co-adapt in response to changes in motor performance during HHI may be particularly relevant to the dynamic nature of rehabilitation. Patient needs for robotic interactions change over time due to fatigue (short-term) and functional improvement in performance (long-term). Thus, rehabilitation robots should ideally be able to engage in this short and long term bi-directional adaptation through haptic interactions alone. Identifying specific changes that occur in the haptic interactions between human partners as a result of fatigue (i.e. need more assistance) or improvements in performance (i.e. needing less assistance) may provide insight into particular characteristics that could be used by rehabilitation robots to identify when and how they should adapt the manner in which they are interacting with a patient to provide an optimal interaction. The ability of rehabilitation robots to engage in such co-adaptation would reflect an ideal form of motor assistance as described by Jarasse [36].

#### How does motor redundancy affect cooperative sensorimotor interactions?

Human movement motor control strategies can often be defined by abstract variables that are not directly encoded by individual sensors or controlled by individual actuators. Rather, they tend to be based on whole limb or whole body characteristics. During reaching, balance, and locomotor tasks, humans have been shown to control abstract motor variables such as endpoint location [95, 96], limb stiffness [97–100], whole-body center of mass dynamics [75, 101], and angular momentum [102] rather than individual joint motions or muscles. Moreover, joint kinematics are more variable than task-level kinematics [103, 104], a relationship that arises due to the redundancy in the neuromuscular system where there is no one-to-one mapping between joint kinematics and task-level kinematics. As a result motor tasks can be performed using a variety of joint configurations.

It may be important to dissociate the control of joints and of abstract motor variables to understand sensorimotor strategies during cooperative HHI and their translation to rehabilitation robotics. Recent studies suggest that sensorimotor recovery is facilitated when variability in the underlying control of joints and muscles is allowed [22, 105, 106]. In contrast, most rehabilitation robots specify joint-level motions [13, 107], although there exist those that only constrain task-level variables [14]. Determining how motor redundancy is used during HHI may inform design and control decisions in rehabilitation robotics. For example, if sensorimotor interaction strategies used by human partners involve constraining joint-level variability to successfully perform a given motor task, robots that take the form of exoskeletons and maintain some level of control over individual joints may be ideal. Alternatively, end-effector types of robots that exert task-level/endpoint control and allow more joint-level variability may encourage more natural and intuitive interactions between patients and robots during rehabilitation. From the perspective of patient safety or confidence, the most appropriate control scheme or robot design may vary over the course of rehabilitation between endpoint and joint-level control. The emergence of differences or changes in how motor redundancy is managed during the performance of joint motor tasks between human partners may yield valuable insight into how we choose to manipulate motor redundancy through HRI for the purposes of motor rehabilitation. Further, within the context of sensorimotor cooperation, motor redundancy may allow participants with dissimilar skill levels or movement patterns, such as patient and therapist, to be paired yet successfully achieve a task-level motor goal.

The need to understand how motor redundancy is used during HHI reinforces the need to identify principles of sensorimotor interaction that are derived from whole-body motor tasks that require multiple degrees of freedom. Therefore the use of more complex whole-body movements that allow for a greater degree of redundancy in task performance deserve greater consideration moving forward. While such motor behaviors can be difficult to analyze, one way to manage the increased complexity that arises from such tasks would be to split up and analyze specific well-defined phases of the task. For example, in a cooperative or collaborative locomotor task one may choose to analyze specific phases such as initiation, and termination or other periods of transition between different locomotor phases that are well defined. The identification of principles of motor interaction based on whole-body movements will be more applicable to robotics in physical rehabilitation, where more often than not, the focus is on complex movement patterns that involve multiple degrees of freedom.

#### How are task-level motor goals communicated through haptic interaction?

While a range of sensory information can be used to inform intentions and actions during cooperative motor tasks, many rehabilitation robots interact primarily with users through haptics (*e.g. lower limb exoskeletons or prostheses*). Similarly, therapist-patient interactions are often driven by haptic interactions. While it is likely that much of the information regarding motor goals can be communicated purely through haptic feedback and dynamic interactions [108], our understanding of those interactions remains limited. This is due in large part to the fact that most of the motor tasks used to study HHI to date have included visual feedback. Additionally, many of the motor goals that have been studied (*e.g. move or pass a small object a short distance, track a visual target*), are rather limited and may not reflect the motor goals that are the focus of physical rehabilitation. By expanding the work on HHI to include motor goals common to rehabilitation (*e.g. stability, maneuverability*), we may develop a better understanding of what force interactions are capable of encoding. In turn this may influence how roboticists design haptic interfaces for rehabilitation robotics. Ultimately, this relies on the selection of motor activities that can be performed with and without the use of vision and that allow for the assessment of motor complex motor goals in order to isolate the influence of haptic interactions.

## Conclusion: moving HHI research forward

Our desire to develop robots that can physically interact with humans in intuitive and biologically inspired ways has revealed a vast field of human-human physical interaction that has only begun to be studied. It may be advantageous to design robots that can be optimized to the functioning of the human nervous system. However, much work is to be done in human-human sensorimotor interactions before any general principles of cooperative sensorimotor control can be firmly established. As a first step, we have identified some areas of potentially fruitful investigation to reveal principles of sensorimotor cooperation in HHI that are directly relevant to the design of physical interfaces and control schemes for rehabilitation robots. Specifically, we propose that new experimental paradigms should be developed that can address open questions of how motor redundancy, varying skill level, specific role assignment, and reliance on haptic feedback play a role in the haptic cues and physical interactions between individuals. In particular, more attention in the specific areas of sensorimotor assistance and sensorimotor education could provide some guidance in the design of haptic interfaces and controllers for rehabilitation robots. Possible motor tasks that could be used in such paradigms include but are not limited to: handshake, partner dance, sawing, carrying objects, leading an individual with visual impairment, and as a more direct clinical task, therapist-patient interactions during rehabilitation. While several of these tasks have served as motivation for a number of HHI studies, we still lack a basic understanding of the magnitude of forces used in these tasks. Additionally, how interaction forces contribute to task performance, or provide a channel for communicating information about motor performance, intent, and skill remains unknown. The identification of general principles of sensorimotor interaction between human partners may also be applicable to an alternative perspective of human-robot interaction in rehabilitation. While the application of HRI in rehabilitation has traditionally focused on the robot-patient interaction, future work may also wish to consider HRI in rehabilitation from the perspective of a robot interacting with a therapist. How might a robot that is focused on the therapist assist him or her in achieving their goals for the patient? While the nature and challenges presented by the interaction between robot and therapist are likely to be different from those of robot-patient interactions, principles derived from the study of HHI may serve to inform such interactions as well. Overall, the identification of guiding principles that drive human-human sensorimotor interactions have the potential to further the design, control and use of rehabilitation robots that can physically interact with humans in intuitive and biologically inspired ways, thereby enhancing rehabilitation outcomes.

## Authors’ original submitted files for images

Below are the links to the authors’ original submitted files for images.
Authors’ original file for figure 2

## Abbreviations

HHI: Human-human interaction
HRI: Human-robot interaction.
