# Supplementary material for: Perspectives on human-human sensorimotor interactions for the design of rehabilitation robots
Source: J Neuroeng Rehabil. 2014 Oct 6;11:142. doi: 10.1186/1743-0003-11-142 (PMC4197261; doi:10.1186/1743-0003-11-142)
Supplement: Supplementary file 1 — Authors’ original file for figure 2 [file 12984_2014_662_MOESM1_ESM.docx]

| **Principal Findings** | | **Papers** |
| --- | --- | --- |
| *Experimental Paradigms* | | |
| 1. HHI research has predominantly focused on sensorimotor collaboration, not cooperation | | |
| *Sensorimotor collaborations* | Reed [56,65], Groten [70], Melendez-Calderon[43], Ikeura[50], Rahman[51], Basdogan[52], Sallnas[53], Gentry[54,64], van der Wel[55], Feth[66] |  |
| *Sensorimotor cooperation* | Ikeura[60], Galvez[86] |  |
| 1. The majority of HHI research has used visuomotor tasks with limited degrees-of-freedom | | |
| *Constrained visuomotor tasks* | Melendez-Calderon[43], Ikeura[50], Rahman[51], Basdogan[52], Sallnas[53], Gentry[54,64], van der Wel[55], Reed[56] |  |
| *Whole-body non-visuomotor tasks* | Galvez[86] |  |
| 1. In most HHI research, specific roles for each member of a dyad are rarely defined ahead of time | | |
| *Unassigned roles* | Reed[65], Groten[70], Melendez-Calderon[43], Rahman[51], Basdogan[52], Sallnas[53], Gentry[54,64], van der Wel[55], Reed[56,65], Feth[66] |  |
| *Assigned roles* | Ikeura[50,60] |  |
| *Experimental Outcomes* | | |
| 1. Dyads typically perform as well or better than either member of a dyad alone | |  |
| *Superior dyad performance* | Reed[65], Gentry[54], Feth[66] |  |
| *Equivalent individual and dyad performance* | van der Wel[55] |  |
| 1. The addition of haptic feedback improves dyad performance compared to visual feedback alone | | Basdogan[52], Sallnas[53], Gentry[64], Groten[67,71] |
| 1. Members of a dyad apply higher forces than during either of their individual performances | | van der Wel[55], Reed[68], Groten[70,71], Feth[66] |
| 1. Members of a dyad spontaneously assume specific roles, performing portions of a joint motor task | | Melendez-Calderon[43], Reed[32, 56] |
| *Starting or ending movement* | Reed [32,68] |  |
| *Adding or absorbing energy* | Feth[66] |  |
|  | |  |
